# Evaluation of the Carbon Sink Capacity of the Proposed Kunlun Mountain National Park

**DOI:** 10.3390/ijerph19169887

**Published:** 2022-08-11

**Authors:** Li Zhao, Mingxi Du, Wei Du, Jiahuan Guo, Ziyan Liao, Xiang Kang, Qiuyu Liu

**Affiliations:** 1 School of Human Settlements and Civil Engineering, Xi′an Jiaotong University, Xi′an 710049, China; 2 Northwest Surveying, Planning Institute of National Forestry and Grassland Administration, Key Laboratory National Forestry Administration on Ecological Hydrology and Disaster Prevention in Arid Regions, Xi’an 710048, China; 3 School of Public Policy and Administration, Xi’an Jiaotong University, Xi’an 710049, China; 4 Co-Innovation Center for Sustainable Forestry in Southern China, College of Biology and the Environment, Nanjing Forestry University, Nanjing 210037, China; 5 Chengdu Institute of Biology, Chinese Academy of Sciences, Chengdu 610041, China; 6 Institute of Environment Sciences, Department of Biology Sciences, University of Quebec at Montreal, Case Postale 8888, Succ. Centre-Ville, Montreal, QU H3C 3P8, Canada

**Keywords:** climate change, nature protected area, management schemes, CMIP6

## Abstract

National parks, as an important type of nature protected areas, are the cornerstone that can effectively maintain biodiversity and mitigate global climate change. At present, China is making every effort to build a nature-protection system, with national parks as the main body, and this approach considers China′s urgent goals of obtaining carbon neutrality and mitigating climate change. It is of great significance to the national carbon-neutralization strategy to accurately predict the carbon sink capacity of national park ecosystems under the background of global change. To evaluate and predict the dynamics of the carbon sink capacity of national parks under climate change and different management measures, we combined remote-sensing observations, model simulations and scenario analyses to simulate the change in the carbon sink capacity of the proposed Kunlun Mountain National Park ecosystem over the past two decades (2000–2020) and the change in the carbon sink capacity under different zoning controls and various climate change scenarios from 2020 to 2060. Our results show that the carbon sink capacity of the proposed Kunlun Mountain National Park area is increasing. Simultaneously, the carbon sink capacity will be improved with the implementation of park management and control measures; which will be increased by 2.04% to 2.13% by 2060 in the research area under multiple climate change scenarios. The research results provide a scientific basis for the establishment and final boundary determination of the proposed Kunlun Mountain National Park.

## 1. Introduction

National parks, as the most important parts of the nature protected areas, are the cornerstones that effectively maintain biodiversity and mitigate global climate change [1,2,3]. Since the first national park, Yellowstone National Park, was established in the United States in 1872 [4], national parks have been widely welcomed by people all over the world. They have been promoted in most countries and regions because they greatly protect a large area of completely natural ecosystems, provide the largest accumulation of biomass and carbon reserves, and make a significant contribution to mitigating the impact of climate change by regulating the carbon cycle [5,6,7]. The establishment of national parks will protect ecosystems from the interference of human activities and the impact of land-use change, so that vegetation and soil can store carbon in the medium and long term [8,9]. These parks can remove carbon dioxide (CO_2)_ from the atmosphere and store it in mineral, organic and ocean reservoirs for a long time, thus mitigating global climate change [10]. Therefore, national parks play an important role in the global carbon cycle. In particular, through effective control, assessment and prediction, they have a very large potential to mitigate CO_2_.

In recent years, relevant scholars have performed many studies on the temporal and spatial characteristics, influencing factors, simulation and prediction, carbon emission policies and other aspects of national park carbon sinks. Zhang et al. used the land ecosystem dynamic model (DLEM) to evaluate the carbon reserves of the Great Smoky Mountains National Park from 1971 to 2001 and analyzed the impact of environmental change on the dynamics of carbon reserves [11]. Cambule et al. evaluated the total amount, spatial distribution and reasons for changes in soil organic carbon in the Limpopo National Park in southwest Mozambique [12]. Tue et al. quantified the carbon storage in the tropical mangrove ecosystem of Mui Ca Mau National Park in Vietnam by measuring trees, roots, fallen sawdust, sediment organic carbon and total depth, as this information can be used to help develop more accurate climate change models and mitigation strategies as well as to effectively reduce emissions from deforestation and degradation (REDD+) schemes [13]. Grossi et al. provided an overall method to quantitatively and qualitatively assess the annual emissions and removal of greenhouse gas (GHG) in the Italian Natural Park, which can help the national major planning strategies to effectively mitigate the greenhouse gas emissions and support the environmental certification process [14]. Dimobe et al. established a research basis for achieving the goals set in the national established contribution and whether an effort helps to maintain or increase the carbon stored in the region by assessing the carbon sink capacity of the national parks. This provides a scientific basis for the sustainable management of the natural ecosystems in other national parks [9]. Peng et al. used partial derivations and division to quantify the contributions of climate change (CC) and human activities (HA) of Qilian Mountain National Park to net primary productivity (NPP) and created six different scenarios. Finally, it was found that the water and soil conservation project and ecological restoration project have made positive contributions to the ecological environment of Qinghai Province [15]. In addition, some studies evaluated the changes in water conservation and carbon sequestration in the Huangshan United Nations Educational, Scientific and Cultural Organization Global Geopark (HUGG) forest during 2000–2015. The relationship between these ecosystem functions and various control factors was analyzed, providing a scientific basis for the sustainable management of natural ecosystems in geoparks and other types of nature protected areas [16]. Especially for China, under the background of promoting the construction of high-quality national parks during global climate change, there is still a lack of systematic research on how the national parks can actively respond to climate change in planning and management. It is necessary to evaluate the carbon sink flux of national parks and analyze its main influencing factors, which will help to promote the active construction of China′s nature reserve system, with national parks as the main body, and achieve the goal of “carbon neutrality”.

The core idea of establishing national parks in this work is “ecological protection first, followed by national representation and public welfare” [17]. The establishment of national parks is outstanding in protecting ecosystems and species diversity and provides a variety of ecosystem services, creating a unique carbon pool foundation [18,19,20]. In 2013, the Third Plenary Session of the 18th Central Committee of the Communist Party of China first proposed establishing a national park system, clarifying the dominant position of national parks in the National Nature Protected Area System, and helping the reform of ecological environment protection and ecological civilization systems. From 2017 to the present, the Chinese government and its competent departments have successively issued the overall plan for the establishment of a national park system; the guiding opinions on the establishment of a nature protected area system with national parks as the main body; the opinions of the Central Committee of the Communist Party of China and the State Council on the complete, accurate and comprehensive implementation of the new development concept to complete a good job in obtaining the carbon peak and achieving carbon neutralization; and the notice of the State Council on printing and issuing the action plan for obtaining the carbon peak before 2030. It further defines the goal, direction and connotation of establishing a nature protected area system in China and integrates the establishment of a number of national parks. By 2030, we will strive to reach the peak value of CO_2_. After reaching the peak value, carbon emissions will decrease steadily, and the ecological environment will gradually improve. We will strive to achieve carbon neutrality by 2060, laying the foundation for the realization of a beautiful China and the construction of ecological civilization [21,22,23]. To date, China has established approximately 12,000 nature protected areas, covering 18% of the national land area [24], and has approved the establishment of a number of national parks, such as Sanjiangyuan. China is a country with rapid population growth, economic growth and urbanization that can better protect its complete natural ecosystem, store a large amount of carbon and play an important role in the regional and global carbon balance by means of establishing national parks.

As the third-class plateau in the world [25], the Qinghai–Tibetan Plateau has a unique global natural and human ecosystem [26,27,28,29]. It is a vulnerable area highly sensitive to climate change and human activities [30], and it is an important ecological barrier in China and even in Asia. As an important geographical unit of the Qinghai–Tibet Plateau, Kunlun Mountain has been listed as a candidate area for the Qinghai Tibet Plateau National Park Group [31,32]. The ecosystem services provided by the proposed Kunlun Mountain National Park will support the economic development of the local herdsmen. However, in recent years, with overgrazing, man-made destruction and the continuous deterioration of the natural environment, the distribution of important ecosystems in the region has been uneven, and the dynamic changes in vegetation productivity have great differences in their spatial patterns. The establishment of the national park and the formation of different management modes will lead to significant changes in vegetation and land use. These changes are expected to affect the growth or reduction in carbon sink capacity in and around the proposed Kunlun Mountain National Park. Therefore, it is both necessary and meaningful to ensure the high-quality construction of national parks in the Qinghai–Tibet Plateau to promote a change in the carbon sequestration from different management and control intensities through the establishment of national parks [33,34].

In summary, to evaluate and predict the dynamics of the carbon sink capacities of national parks under climate change and different management measures, this study used the proposed Kunlun Mountain National Park as the research object, evaluated the carbon sequestration capacity of the proposed Kunlun Mountain National Park in different periods, and analyzed the driving factors affecting the carbon sequestration capacity in this region to determine the impact of different control intensities on the carbon sequestration flux change in the proposed Kunlun Mountain National Park. In addition, we put forward some opinions on how to manage the carbon sink capacity and improve ecological protection in the region, and we provide suggestions for future high-quality development. The research questions to be discussed in this study are as follows:

1. What was the size, change and spatial distribution of the carbon sink capacity when there was no national park in the study area over the past 20 years?
2. What are the main driving forces and influencing factors behind the change in the carbon sink capacity?
3. For different climate scenarios in the future, how will the carbon sink capacity of the national park area change? If the national park is established, can the implementation of the control measures help to improve the carbon sink capacity of the research area?

## 2. Materials and Methods

### 2.1. Study Area

**Figure 1:**
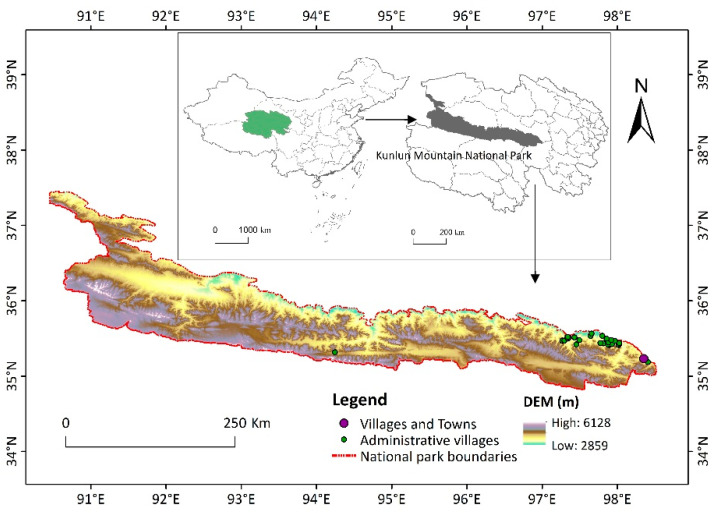
The geographic location of the study area.

The study area was the proposed area of Kunlun Mountain National Park, which is a geographical subunit (90°31′03″~98°34′13″ E and 35°15′21″~37°52′18″ N) (Figure 1), located on the northern edge of the Qinghai–Tibet Plateau in northwest China, including the Kunlun Mountains and the Qaidam Basin. It is a strategic fortress and an important transportation hub connecting Xinjiang, Tibet and Gansu in western China. This area includes 1 prefecture-level city, 3 counties (cities), 9 towns and 25 administrative villages. The park covers a total area of 59,519.22 km^2^. There are two original nature reserves included in the study area: Mt. Kunlun Geopark and Mount Kunlun UNESCO Global Geopark. The proposed Kunlun Mountain National Park area has many geographical landforms, such as plateau meadows, glaciers and deserts, as well as rich natural landscape resources and wild animal and plant resources, such as the Tibetan antelope, Tibetan wild ass and angiosperm herbs unique to the Qinghai–Tibet Plateau [35]. The altitude rises gradually from east to west, ranging from 2800 m to 6200 m, with an average altitude of approximately 4500 m. The regional ranges of annual precipitation and annual average temperature are 100~5500 mm and below 0 °C, respectively. Due to the high altitude and low temperature, the air in most of the areas is thin, and the plant growth cycle is short.

The land use data of the Qinghai Lake Basin came from the vector data of the third land survey of the Qinghai Provincial Department of Natural Resources in 2022. Referring to the classification of land use status and the land classification of the third national land survey, the land use types in the study area were classified and counted. The digital elevation model (DEM) data were derived from the geospatial data cloud (http://www.gscloud.cn, (accessed on 1 March 2022)) with a spatial resolution of 30 m × 30 m. Other related information on the climate and human activity factors can be found in Table 1 and detailed described in following sections.

### 2.2. Simulation of Carbon Sink Capacity

Since the vegetation in the Kunlun Mountains is dominated by grassland and the grassland vegetation is annual herbaceous plants, the annual net primary productivity (NPP) value of grassland vegetation can be regarded as the vegetation carbon sink capacity [36]. The NPP data from the Moderate Resolution Imaging Spectroradiometer (MODIS) were used to estimate the vegetation carbon sink capacity in the Kunlun Mountains. Specifically, we collected the MOD17A3HGF V6 product (https://doi.org/10.5067/MODIS/MOD17A3HGF.006, (accessed on 10 March 2022)), which provides information about annual NPP at a 0.5 km pixel resolution over the period of 2000–2020. This annual NPP was derived from the sum of all of the 8-day net photosynthesis (PSN) products (MOD17A2H) from a given year. This product has been widely applied in the research on vegetation and carbon dynamics [37,38].

To explore the relationship between the carbon sink capacity and environmental and human activities, we collected the soil information of the study area, including soil organic carbon content (SOC), pH, soil bulk density (BD), soil total nitrogen (TN), soil total potassium (TK) and soil volumetric water content (VWC), from a global soil dataset of NASA (The National Aeronautics and Space Administration) [39] at a 1 km pixel resolution.

Recently, the state-of-the-art climate models participating in Phase Six of the Coupled Model Intercomparison Project (CMIP6) have become available for the public and can be used for climate simulations [40]. To better evaluate the carbon sink changes under future climate change, four shared socioeconomic pathways (SSPs), drawn from Tier 1 of ScenarioMIP: SSP1-2.6 (+2.6 Wm^−2^ imbalance; low forcing sustainability pathway); SSP2-4.5 (+4.5 Wm^−2^; medium forcing middle-of-the-road pathway); SSP3-7.0 (+7.0 Wm^−2^; medium- to high-end forcing pathway) and SSP5-8.5 (+8.5 Wm^−2^; high-end forcing pathway), were selected as the reference scenario to obtain the corresponding climate data. The climate data, including the mean annual temperature (MAT) and mean annual precipitation (MAP), were also collected from WorldClim [41] at a 1 km pixel resolution. The aridity index (AI) and potential evapotranspiration (PET) were collected from the Consortium for Spatial Information (CGIAR-CSI; http://www.cgiar-csi.org, (accessed on 10 March 2022)) at a resolution of 1 km.

We derived the human footprint index (HFI, 1 km × 1 km) from the Earth Observation System Data and Information System [42] as an indicator to evaluate the impacts of human activity on the carbon sink capacity in the study area. The NPP data were resampled to a 1 km pixel resolution to match the resolution of the climate and human activity data. More detailed information about the climate and human activity factors used in this study can be found in Table 1.

### 2.3. Correlation of Carbon Sink Potential with Human Activities and Natural Sensitive Elements

The generalized linear mixed effects models (GLMMs) [43] have been widely used in ecological research to classify related complex problems affected by multiple factors [44]. The advantages of using GLMMs include the following: (1) multicollinearity between the spatial and temporal scales is reasonable in all of the models; (2) random effects can be included and (3) the interaction terms between the spatial and temporal scales can be easily included in the model [45]. The general statistical model was yi = µ + Si + Ti + Si × Ti + ei + ϵi, where yi is the scaling exponent b from curve fitting; Si, Ti and Si × Ti are the fixed effects of the spatial scale, temporal scale, and their interactions, respectively; ei is the random effect with which each experiment was associated with independent errors following a normal distribution; and ϵi is the residual error [43]. In this study, the GLMMs were used to quantify the effects of the climate indicators and human activities on the vegetation carbon sink capacity. In parallel, this model was used to identify the indicators that could significantly affect (*p* < 0.05) the carbon sink capacity and was used to predict the changes in the carbon sink capacity under different management scenarios.

First, a multi-collinearity test, using Pearson’s correlation, was conducted to determine the relationship between the independent variables (environmental variables) indicated by the results (i.e., NPP) matrix, and the variables with correlation coefficients greater than 0.8 were removed [46]. Second, the relationships between NPP (dependent variable) and the environmental variables (independent variables) were quantified, using the mixed effect models using the lmer function in the R package ‘lme4’ [47], which fits the models based on a restricted maximum likelihood. Third, after model screening using the dredge function in the ‘MuMIn’ R package [47], the best model was obtained, as that included the environmental variables that explained the most variation in the NPP. Finally, using the predict function within the ‘lme4’ R package, the future NPP was then predicted. For the best linear mixed model, we calculated the R^2^ value using the ‘MuMIn’ R package. R^2^_marginal_ denotes the variance explained by the fixed effects (i.e., climatic, soil factors and HFI), and R^2^_conditional_ is the variance explained by both the fixed and random effects (i.e., sites). All of the analyses were conducted in R 4.0.4 software (R Core Team, 2021. R: A language and environment for statistical computing. R Foundation for Statistical Computing, Vienna, Austria).

### 2.4. Zoning Control Path of the National Park

**Figure 2:**
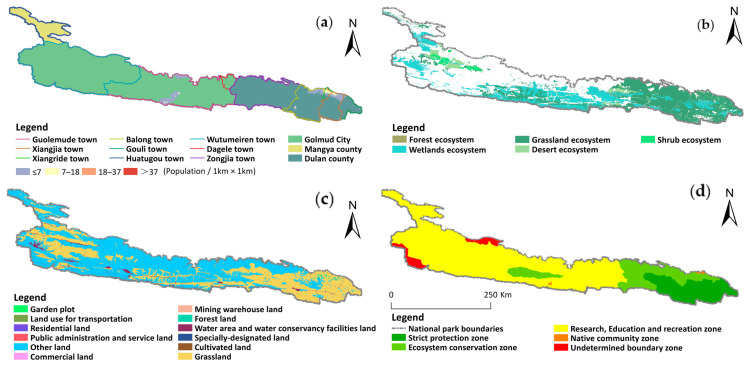
Functional zone map of the proposed Kunlun Mountain National Park. (**a**) Population distribution and administrative zoning map; (**b**) Ecosystem map; (**c**) Land use status map; (**d**) Functional zoning scheme.

To comprehensively reflect the spatiotemporal pattern and future scenario changes of the carbon sink capacity in the proposed Kunlun Mountain National Park, it is necessary to clarify which places could be protected and utilized appropriately; it is also necessary to avoid conflict between economic development and ecological protection, use carbon sink information to more scientifically promote economic construction, and consider how economic construction can better protect carbon sink growth. Therefore, we focused on the differentiated functional zoning of the national parks from the perspective of carbon sinks. Additionally, the overall plan for the reform of the ecological civilization system clearly stipulates that the national parks are stricter than other types of natural reserves [48]. Their primary function is to protect the authenticity and integrity of important natural ecosystems. They also have comprehensive functions, such as scientific research, education and recreation [49]. We further weighed the carbon sink capacity, ecosystem, population distribution and land use type in 2020 for functional zoning. According to the code for the functional zoning of national parks, the study area was divided into strict protection areas, ecological conservation areas, science and education recreation areas and traditional utilization areas [50]. In addition, the areas with low or no carbon sink capacity, low or no landscape resources, and low or no ecosystem integrity were classified as boundary undetermined areas (Table 2, Figure 2).

### 2.5. Estimation of the Future Carbon Sink Capacity under Different Climate Change Scenarios

The establishment of national parks focuses on protecting the authenticity and integrity of the important regional ecosystems, which is critical to the growth of carbon sinks. Therefore, we assessed the carbon sequestration capacity of the future national parks by constructing virtual scenarios of different zoning controls and climate change [51]. Our scenario settings included the following scenario settings:

1. Scenario A: Scenario A refers to the change in carbon sink capacity due to natural climate change by 2060. The setting of this scenario included the following indicators: MAP indicators, which refer to mean annual precipitation; and MAT indicators, which refer to mean annual temperature. Here, we estimated the change in carbon sink capacity by obtaining the MAP and MAT data of four representative concentration pathways (RCPs), RCP2.6, RCP4.5, RCP7.0 and RCP8.5 climate change scenarios of SSPs 1, 2, 3 and 5;
2. Scenario B: Scenario B refers to the change in carbon sink capacity due to the intensity of human activities under the premise of different zoning controls by 2060. The setting of this scenario included the following indicators: the setting of the meteorological data was consistent with scenario A. The HFI indicators refer to the human footprint index. The setting of such indicators will lead to different levels of human activity factors due to the difference in the regional control levels. Here, we believe that under the premise of different zoning controls, the proportion of human activity intensity will be reduced from high to low. The strictly protected areas will be reduced by 100%, the ecological conservation areas will be reduced by 75%, the scientific and technological recreation areas will be reduced by 50%, the traditional utilization areas will be reduced by 25% and the areas with undetermined boundaries will be reduced by 0%.

By comparing the relevant results of scenarios A and B, this work discusses the impact of the relevant control measures after the establishment of the national parks on the carbon sink capacity under different climate change scenarios.

## 3. Results and Analyses

### 3.1. Temporal and Spatial Trends in the Carbon Sink

#### 3.1.1. Characteristics of Temporal Variation

**Figure 3:**
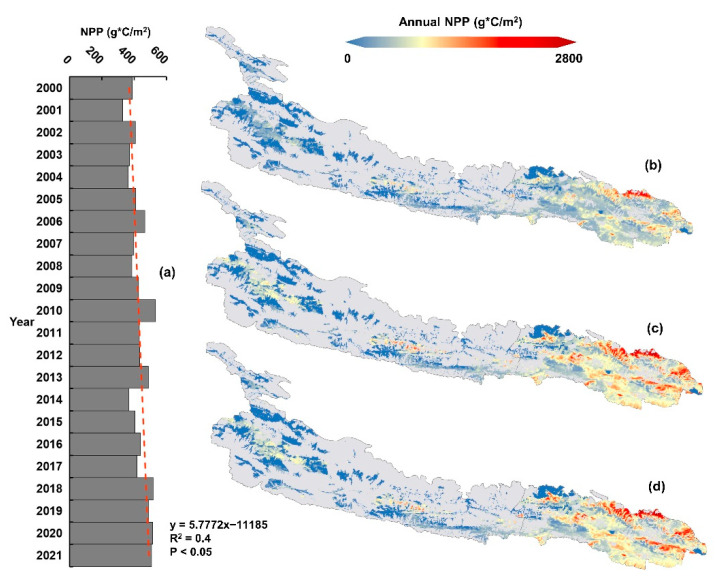
Annual average NPP of the vegetation area over the study area from 2000 to 2021 (**a**) and NPP spatial distributions in 2000 (**b**); 2010 (**c**) and 2020 (**d**). NPP is net primary productivity.

From 2000 to 2020, the total carbon sequestration of the proposed Kunlun Mountain National Park continued to increase (Figure 3a). However, there were fluctuations between the years, showing a convergence trend overall, but still far from obtaining the peak carbon level. In 2000, the vegetation coverage was low, the climate was dry and rainless, and the average vegetation carbon sink was 386 g*c/m^2^/year. In 2010, with the increase in precipitation and the decrease in temperature in the study area, the improvement in climate conditions increased the carbon sink of the vegetation and removed the carbon consumed by soil respiration, and the vegetation played a role in the carbon sink total. In 2010, it reached 529 g*c/m^2^/year, an increase of nearly 37%. In 2020, although the total amount of vegetation carbon sequestration increased compared with that in 2000, due to the gradual impact of human activities, the total amount of vegetation carbon sequestration decreased slightly compared with that in 2010, reaching 513 g*c/m^2^/year. Overall, the carbon absorption of the region has gradually increased, and the carbon sink capacity has been enhanced, reflecting an improvement in the vegetation status and in the ecological environment in the region. However, in the state of natural growth and development, without national park construction and without any policy control, the growth rate would be limited and unstable. 

#### 3.1.2. Characteristics of Spatial Distribution

From the perspective of the spatial distribution of carbon sinks in the proposed Kunlun Mountain National Park (taking 2000, 2010 and 2020 as the examples; Figure 3b–d), the carbon sinks in the study area showed a spatial pattern of continuous outward expansion from 2000 to 2020, showing different regional change trends. From the perspective of the total carbon sink, the carbon sink in the proposed Kunlun Mountain National Park roughly presented a pattern of being “high in the east and low in the west” or “Northeast > East > Central > West”; low carbon sink value areas were mainly concentrated in the northwest of the proposed Kunlun Mountain National Park, northeast and south of Huatugou town, Mangya city, and south of Wutumiren Township, Golmud city. These areas were mainly other land types (bare land), and there was no natural ecosystem. With the gradual warming and wetting of the climate conditions, the function of the regional carbon sink will decline. High carbon sink value areas were mainly concentrated in the north of Balong Township, the north of Xiangride Town, the middle of Xiangjia Township and the north of Gouli Township in Dulan County. These areas are located in the Hehuang valley of the Qinghai Plateau and its surrounding areas. The altitude is low, the temperature is appropriate, the water sources are plentiful and the carbon sink capacity of the vegetation is increasing each year.

From 2000 to 2010, the spatial pattern of the carbon sink changed. The central part of Golmud town, Golmud city, the northern part of Balong Township in Dulan County, the northern part of Xiangride Town, the central part of Xiangjia Township and the northern part of Gouli Township all rebounded. From 2010 to 2020, carbon sequestration decreased in the middle of Golmud town, the middle and south of Zongjia Town, Dulan County and the north of Gouli Township. Overall, the total carbon sink amounts in Xiangride town and Xiangjia Township of Dulan County in the proposed Kunlun Mountain National Park increased significantly, while the carbon sink amounts in Wutumiren Township and Golmud town of Golmud city showed a downward trend.

### 3.2. Drivers of Carbon Sink Capacity

Referring to the existing research results and considering the availability of the data, this work focused on the impact of six factors on the carbon sink capacity, to better understand the relationship between the carbon sink capacity and driving factors, including the BD, HFI, MAP, MAT pH and potential evapotranspiration (PET). Based on the GLMM method, we obtained the relationship between the relevant environmental variables and human activity factors and vegetation carbon sink capacity (Table 3). From the influence degree of each factor, the BD, HFI, MAP, MAT, pH, TK, TN, TP and VWC were highly significantly correlated with the carbon sink capacity. Among them, the BD, MAP, MAT, pH and VWC were significantly positively correlated with the improvement of the carbon sink capacity, showing that the influence of precipitation, air temperature and soil water content on vegetation growth in the study area was mainly a coupling effect, and they acted on vegetation growth together. The TK, TN, TP and HFI were negatively correlated with the carbon sink capacity. This result indicates that the increase in related soil trace elements and the accumulation of some human activities hindered the accumulation of carbon sinks. This is due to grazing in the area with human activities, and excessive fertilization, production and living activities have produced excessive carbon emissions and affected the growth of the ecosystem in the area.

### 3.3. Functional Zoning of National Parks

In addition, to further promote the establishment of the proposed Kunlun Mountain National Park and achieve the goal of carbon neutrality, we conducted preliminary national park functional zoning and national park boundary selection from the perspective of carbon sinks, to clearly specify the protection level and other utilization areas of carbon sinks on each plot in the national park [52]. We divided the functions of the proposed Kunlun Mountain National Park into five parts (Figure 2d). The strictly protected area covers 8401.72 km^2^, accounting for 14.12% of the total area of the park. It retains the original forest and grassland ecosystems and the areas with the highest carbon sink benefit, provides suitable habitats for important organisms and is the key function of the ecosystem services.

The ecological conservation area covers a total area of 9311.61 km^2^, accounting for 5.64% of the total area. It is an important and fragile grassland system, including the original habitat or area of relatively large patches that have been damaged and need natural restoration. Generally, this area is strictly protected by ecological barriers and buffer zones. The traditional utilization area is 145.8 km^2^, accounting for 0.24% of the total park, mainly distributed in western Yaochi, Golmud town, Golmud city, northern Xiangride town, Dulan County and northern Balong Township. There are many herdsmen in this area, and the ecosystem has a strong ecological service capacity. Therefore, the area is suitable for traditional community production and management activities and traditional grazing activities.

The science and education recreation area covers an area of 39,555.4 km^2^, accounting for 66.46% of the total area. This area is rich in landscape resources and has convenient transportation. It is a concentrated area of unique landscape resources and geological resources, such as the Yaochi and Yuxu peaks and an earthquake fault zone. In this area, activities, such as sightseeing and entertainment, science education and franchising, can be promoted to show the natural and cultural ecological landscape of the park. In addition, we assessed the area with an undetermined boundary, which had an area of 2104.69 km^2^, accounting for 3.54% of the total area, providing a scientific basis for the next boundary delimitation of the proposed Kunlun Mountain National Park (Figure 2d).

### 3.4. Carbon Sink Capacity Prediction under Different Scenarios

**Figure 4:**
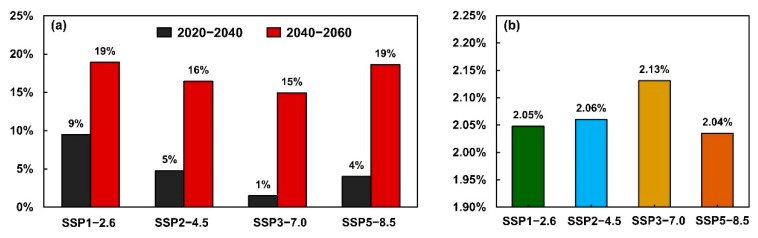
The change rate of the carbon sink capacity in 2040 and 2060 under different climate change scenarios compared with the average carbon sink capacity of the period of 2000–2020 (**a**); The change ratio of carbon sink capacity with management compared with that without management in 2060 under different climate change scenarios (**b**).

**Figure 5:**
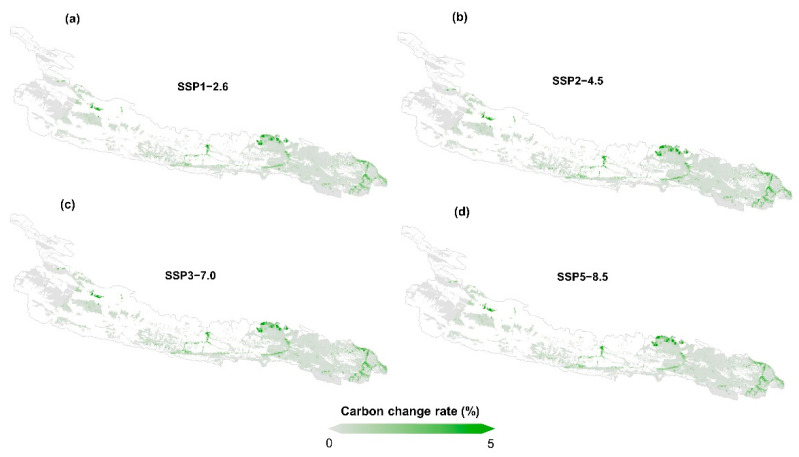
The distribution of the change rate of the carbon sink capacity with and without management in 2060 under climate change scenarios SSP1-2.6 (**a**); SSP2-4.5 (**b**); SSP3-7.0 (**c**) and SSP5-8.5 (**d**).

The carbon sink changes under the different scenarios were formulated to determine the threat of future human activities and climate impacts. Therefore, we summarized the change characteristics of the carbon sink capacity under different scenarios. The result analysis of scenario A showed that, compared with the average carbon sink capacity from 2000 to 2020, the overall average carbon sink capacity of the proposed Kunlun Mountain National Park in 2020–2040 and 2040–2060 improved (Figure 4 and Figure 5). Specifically, during 2020–2040, the capacity increased by 9%, 5%, 1% and 4% under the SSP1-2.6, SSP2-4.6, SSP3-7.0 and SSP5-8.5 scenarios, respectively. During 2040–2060, the carbon sink capacity under each scenario increased more significantly, by 19% (SSP 1-2.6), 16% (SSP 2-4.5), 15% (SSP 3-7.0) and 19% (SSP-8.5). With the establishment of the national park, the level of zoning management and control has continuously improved, and the public′s awareness of sharing has continuously improved. The results based on scenario B showed that the overall improvement in the carbon sink in the study area is obvious. Specifically, we quantified the impact of the implementation of control measures on carbon sink capacity under the future climate change scenarios in the study area where there is human interference. The results showed that the average carbon sink capacity during 2020–2060 increased by 2.05% under SSP1-2.6, 2.06% under SSP2-4.5 and 2.13% under SSP3-7.0, compared with that in 2000–2020.

However, it increased by 2.04% under SSP5-8.5 (Figure 4b). The implementation of control measures has increased the average carbon sink capacity of each climate change scenario by 2.06%. This average increase makes the overall cumulative carbon sequestration in the region reach nearly 83% of the initial year during 2020–2060. In general, the results of scenario B and scenario A showed that under the future climate change scenarios, the control measures related to the establishment of the park will enhance and improve the carbon sink capacity of the study area. This means that the establishment and control of the national parks is practical for the improvement of the carbon sequestration in the study area.

## 4. Uncertainty and Limitations

Due to the spatial heterogeneity of the ecosystems, the response of vegetation to climate factors has different spatial patterns and lag times [53]. Therefore, in future research, the model and parameters should be further adjusted and improved to produce results that are more representative of the actual situation. In addition, the analysis results of the GLMMs on the historical data (2000–2021) (Table 3) showed that human activities could have a significant negative impact on the carbon sink capacity. However, due to the low proportion of population distribution in the study area (3%, HFI > 5), the absolute impact of the relevant control measures on the carbon sink capacity reflected in the results of scenario B may be small.

The particularity of the basic situation in the study area (such as the low proportion of population distribution) is the main reason for this result. In addition, the carbon sink capacity is affected by human society and ecological environmental factors. There may be a complex nonlinear relationship between the carbon sink capacity and various factors. However, the GLMM model has a poor performance in capturing the potential nonlinear relationships. Therefore, there may be some uncertainty in the correlation analysis and prediction results based on the GLMMs. Furthermore, we did not focus on the strict protection area accounting for more than 50% of the park area in the code for the functional zoning of national parks, because we focused on the zoning and prediction from the carbon sink distribution pattern to provide a reference and basis for the final boundary delimitation and zoning of the national park. Of course, we will actively study the boundary delimitation and zoning of Kunlun Mountain National Park in the next step.

## 5. Discussion

According to the temporal and spatial patterns of carbon sequestration in the proposed Kunlun Mountain National Park, with local human activities and without any control policy support, relying on its natural development, the growth rate from 2000 to 2020 was relatively flat. By 2040 and 2060, its carbon sink capacity will increase steadily under the various climate change scenarios. It was clear (Figure 2 and Figure 3) that the main source of the carbon sink capacity was regional forestland, followed by natural grassland. The contribution of bare rock/gravel land to the carbon sink capacity was very small, while the contribution of bare land was almost negligible. After the establishment of the proposed Kunlun Mountain National Park, its incorporation into the national park system as the main nature reserve system can reduce these impacts [54]. Through overall planning, the areas with a high spatial distribution of the carbon sink capacity of forestland and natural grassland should be strictly protected. Additionally, we should strengthen the protection of bare rock/gravel and bare land, incorporate the improvement of carbon storage and carbon absorption functions into the ecological protection and restoration projects, strengthen the protection of the identified areas with high carbon sinks and carbon reserves and prioritize the ecological restoration of the areas with high carbon sink promotion potential to increase the carbon size capacity.

In China, the attitude of local residents toward ecosystem protection can significantly affect the success of conservation measures [55,56]. Through the analysis of the driving factors of carbon sink capacity, we found that human activities have a great impact on the carbon sink capacity. Considering human activities alone produced a significant negative correlation and even became the main driving factor [57]. Of course, as shown in Figure 1 and Figure 3, some of the areas with a high carbon sequestration capacity also had villages and human activities. Therefore, how to balance the protection and utilization of the areas with high human activities and areas with a high carbon sequestration capacity is a future focus of consideration. We suggest that the national park management department issue relevant policy support and financial support to severely eliminate the illegal acts that damage forests and grasslands in areas with a high carbon sequestration capacity, abolish mining rights and hydropower development enterprises, and promote the relocation of ecological immigrants or the control of restricted grazing areas [15] to better promote the integrated development of the national parks and communities and promote the realization of China’s “double carbon goals”.

Climate change is also an important factor affecting the vegetation dynamics and carbon sinks, as has been confirmed by previous studies [58,59]. From the GLMM results (Table 3), there was a general positive correlation between temperature, precipitation and carbon sink capacity in the study area. More precipitation will promote the recovery of vegetation. The increase in the temperature can prolong the growth season of vegetation, especially in arid and semiarid areas, because the available water and solar radiation-enhanced photosynthesis are critical factors [60,61]. However, the plateau ecosystem is very sensitive to changes in climate factors, such as precipitation and temperature. Our research (such as the results of scenario A) captured the impact of future climate change on the growth of vegetation and carbon sink capacity of the proposed Kunlun Mountain National Park, showing that the overall carbon sink capacity will increase under future climate change, but there are differences between scenarios. In addition, the implementation of control measures has different impacts on the carbon sink capacity in the study area under various climate change scenarios. These results further reflect the importance of setting scientific and reasonable control measures for the study area in the future to enhance the carbon sink capacity.

## 6. Conclusions

The main purpose of establishing the national parks in China is to protect the ecological environment, biodiversity and natural resources, protect the integrity and authenticity of typical and unique ecosystems from damage, and leave a valuable natural heritage for future generations [62]. Therefore, this study attempted to predict the change in the carbon sink capacity in the study area in the future by setting the promotion ratio of different regions after the establishment of the national park to promote the carbon neutrality. Our results show that the establishment and construction of the national parks increases the carbon capture and carbon storage capacity [20]. In particular, after the establishment of the national parks, the types, scope and management institutions of the protected areas can be defined, and relevant protection and utilization policies can be formulated to cope with the changes brought about by human activities and the natural climate.

## Figures and Tables

**Table 1:** Information on the climate and human activity factors derived from 13 global spatial layers detailed in the Section 2.

Variable	Units	Source	Origin Spatial Resolution
HFI		EOSDIS (The Earth Observing System Data and Information System)	1 km
Elevation	m	WorldClim	1 km
MAT	°C	WorldClim	1 km
MAP	mm		1 km
AI		Consortium for Spatial Information(CGIAR-CSI)	1 km
PET	mm		1 km
SOC	%	The Global Soil Dataset for Earth System Modeling (GSDE)	1 km
pH (H_2_O)			1 km
BD	g cm^−3^		1 km
TN	%		1 km
TP	%		1 km
TK	%		1 km
VWC	%		1 km

MAT: mean annual temperature; MAP: mean annual precipitation; AI: aridity index; PET: potential evapotranspiration; BD: soil bulk density; TN: soil total nitrogen; TP: soil total phosphorus; TK: soil total potassium; VWC: soil volumetric water content; HFI: human footprint index.

**Table 2:** Functional zoning types of the proposed Kunlun Mountain National Park.

Partition Type	Carbon Sink Capacity	Population Distribution	Ecosystem	Management and Control Requirements
Strictly protected area	High	Nothing	Complete	The natural ecological geographical units, such as the intact original forest ecosystem and alpine meadow ecosystem, are protected in this area. Human activities are strictly prohibited.
Ecological conservation area	Higher	Lower concentration	Relatively complete	This area contains important and fragile ecosystems, which need to be restored to the degraded natural ecosystems, or the influence of external interference must be isolated or slowed in the strictly protected areas. Human activities in principle are restricted.
Science, education and recreation area	Middle	Moderate concentration	Moderately complete	This area has good recreational resources, a cultural landscape and a pleasant environment, and it is convenient to implement a natural experience, eco-tourism, rest and health activities, and moderate human activities.
Traditional utilization area	Lower	Higher concentration	Lower integrity	This area is the production and living space of the original residents. To ensure the basic living needs of the original residents, the urban and rural construction land is strictly controlled in accordance with the overall land use plan. The use is limited in principle.
Boundary undetermined area	Nothing	Nothing	Nothing	There are no important natural resources, unique landscape resources or human activities in this area.

**Table 3:** Statistics for the best model of the relationships between the various drivers and net primary production. Model estimates (mean coefficient estimates) with a 95% confidence interval (CI) and the model variation explained by the fixed effects alone (marginal R^2^) and by both the fixed and the random effects (conditional R^2^) are presented. Values exceeding the significance level are indicated in bold (*p* < 0.05).

	NPP			
Predictors	Estimate	CI	Sum of Squares	*p*
**Fixed effects**				
(Intercept)	−267.88	−446.14–89.62		**0.003**
BD	120.65	18.60–222.70	2.90 × 10^5^	**0.020**
HFI	−2.08	−3.97–−0.19	2.53 × 10^5^	**0.031**
MAP	2.65	2.61–2.70	6.82 × 10^8^	**<0.001**
MAT	23.36	18.25–28.27	4.47 × 10^6^	**<0.001**
PET	0.05	0.03–0.06	1.84 × 10^6^	**<0.001**
pH	43.98	31.72–56.24	2.67 × 10^6^	**<0.001**
TK	−99.42	−113.23–−85.62	1.08 × 10^7^	**<0.001**
TN	−94.72	−174.78–−14.65	2.90 × 10^5^	**0.020**
VWC	−499.30	−803.25–−195.35	5.60 × 10^5^	**0.001**
TP	−141.69	−418.87–135.49	5.42 × 10^4^	0.316
**Random effects**				
σ^2^	54,018.18			
τ00 _Elevation_	46,059.79			
ICC	0.46			
N _Elevation_	1342			
Observations	34,028			
Marginal *R*^2^	0.374			
Conditional *R*^2^	0.662			

NPP: net primary production (g cm^−2^ year^−1^); BD: soil bulk density (g cm^−3^); HFI: human footprint index; MAP: mean annual precipitation (mm); MAT: mean annual temperature (°C); TK: soil total potassium (%); TN: soil total nitrogen (%); TP: soil total phosphorus (%); VWC: soil volumetric water content (%); PET: mean annual potential evapotranspiration (mm).

## Data Availability

All data and computer codes generated during this study are available from the corresponding authors upon reasonable request.
